# Fatty Liver Disease and Non-Alcoholic Fatty Liver Disease Worsen the Outcome in Acute Pancreatitis: A Systematic Review and Meta-Analysis

**DOI:** 10.3390/jcm9092698

**Published:** 2020-08-20

**Authors:** Szilárd Váncsa, Dávid Németh, Péter Hegyi, Zsolt Szakács, Péter Jeno Hegyi, Dániel Pécsi, Alexandra Mikó, Bálint Erőss, Adrienn Erős, Gabriella Pár

**Affiliations:** 1 Institute for Translational Medicine, Medical School, University of Pécs, 7624 Pécs, Hungary; vancsa.szilard@pte.hu (S.V.); davidsum96@gmail.com (D.N.); hegyi2009@gmail.com (P.H.); szaki92@gmail.com (Z.S.); drdunajskastreda@gmail.com (P.J.H.); daniel.pecsi1991@gmail.com (D.P.); miko.szandi@gmail.com (A.M.); dr.eross.balint@gmail.com (B.E.); 2 János Szentágothai Research Centre, University of Pécs, 7624 Pécs, Hungary; adriennhat@hotmail.com; 3 Centre for Translational Medicine, Department of Medicine, University of Szeged, 6725 Szeged, Hungary; 4 Division of Gastroenterology, First Department of Medicine, Medical School, University of Pécs, 7624 Pécs, Hungary; 5 Heim Pál Children’s Hospital, 1089 Budapest, Hungary

**Keywords:** acute pancreatitis, fatty liver disease, non-alcoholic fatty liver disease, hepatology, pancreatology, prognosis

## Abstract

The prevalence of fatty liver disease (FLD) and that of non-alcoholic fatty liver disease (NAFLD) share some risk factors known to exacerbate the course of acute pancreatitis (AP). This meta-analysis aimed to investigate whether FLD or NAFLD carry a higher risk of untoward outcomes in AP. In accordance with PRISMA guidelines, we performed a systematic search in seven medical databases for cohort studies that compared the outcomes of AP for the presence of FLD or NAFLD, and we calculated pooled odds ratio (OR) or weighted mean difference (WMD) with 95% confidence interval (CI). We included 13 articles in our meta-analysis. AP patients with FLD were more likely to die (5.09% vs 1.89%, OR = 3.56, CI = 1.75–7.22), develop severe AP (16.33% vs 7.87%, OR = 2.67, CI = 2.01–3.56), necrotizing pancreatitis (34.83% vs 15.75%, OR = 3.08, CI = 2.44–3.90) and had longer in-hospital stay (10.8 vs 9.2 days, WMD = 1.46, OR = 0.54–2.39). Patients with NAFLD were more likely to have severe AP and longer hospital stay. Both FLD and NAFLD proved to be independent risk factors of a more severe disease course (OR = 3.68, CI = 2.16–6.29 and OR = 3.39, CI = 1.52–7.56 for moderate/ severe vs. mild AP, respectively). FLD and NAFLD worsen the outcomes of AP, which suggests that incorporating FLD or NAFLD into prognostic scoring systems of AP outcomes might improve the prediction of severity and contribute to a more individualized patient care.

## 1. Introduction

Fatty liver disease (FLD) is becoming increasingly common in the Western world, affecting about 25% of the population globally [1]. FLD is a clinicopathologic entity with a histological spectrum that includes simple steatosis and steatohepatitis, also it encompasses a broad variety of etiology. The most common causes of FLD are non-alcoholic fatty liver disease (NAFLD) associated with metabolic syndrome (MetS), alcohol abuse alone or in association with hypertriglyceridemia, and the combination of the causes above. It is widely known that there is a bidirectional association between NAFLD and components of MetS [2]. The presence of NAFLD increases the risk of cardiovascular diseases, type 2 diabetes mellitus, chronic kidney disease, liver cirrhosis, and liver cancer [3].

Acute pancreatitis (AP) is a common acute gastrointestinal disease, posing a substantial social and economic burden [4]. Although the mortality of AP has been decreasing in the past decades, it is still between 2–5% and it remains high, up to 15–25% in subgroups of patients with severe AP, depending on the extent of necrosis and systemic complications [5].

Based on the guidelines issued by the International Association of Pancreatology (IAP) and the American Pancreatic Association (APA), on admission of patients with AP, a three-dimensional approach is recommended for predicting the outcome of AP, combining host risk factors, clinical risk stratification and response to initial therapy [6]. Several prognostic tools have been developed for the early prediction of severe AP and mortality, based on demography, clinical signs and symptoms, laboratory studies and imaging, composing numerous scoring systems (e.g., Bedside Index of Severity in Acute Pancreatitis—BISAP [7], 48 h Acute Physiology and Chronic Health Evaluation—APACHE II score [8], Ranson scores [9], Computed Tomography Severity Index—CTSI [10]). A recent meta-analysis showed that scoring systems have comparable diagnostic accuracy to predict severe AP with area under the curve ranging from 0.73 to 0.83 [11].

The presence of MetS is a proven risk factor of severe AP [12,13]. Pre-existing diabetes mellitus negatively influences the outcome of AP and increases the risk of renal failure, local complications, intensive care compared with the non-diabetic group [14]. Obesity is another risk factor in AP; obese patients have a three-fold increased risk of mortality compared to those with a BMI < 30 [15]. High triglyceride level is also a risk factor, serum triglyceride level higher than 5.6 mmol/L significantly increases the mortality rate (OR = 2.75, 95% CI = 1.28–5.92, *p* < 0.01) [16]. An experimental study in rat AP model demonstrated that the presence of FLD increased pro-inflammatory cytokine production, which may worsen the course of the disease [17]. Cross-sectional studies confirmed that AP is often accompanied by FLD, with a prevalence between 18–43% [18,19].

Since FLD or NAFLD is common in diabetes or obesity worsening the course of AP, it may also act as a potential risk factor in AP. This meta-analysis aimed to investigate whether FLD or NAFLD is associated with a less favorable disease course in AP.

## 2. Methods and Materials

Our study is reported according to the Preferred Reporting Items for Systematic Reviews and Meta-Analysis (PRISMA) 2019 Statement [20]. The study protocol was registered in PROSPERO under registration number *CRD42019123416* (see *https://www.crd.york.ac.uk/prospero*).

### 2.1. Literature Search

A systematic literature search was performed in seven medical databases (PubMed, EMBASE, Web of Science, CENTRAL, WHO global health library, Scopus, and ClinicalTrial.gov) from inception to 13th of November 2019 with the query *pancreatitis AND (“fatty liver” OR FLD OR NAFLD OR steatohepatitis OR steatosis)*. We used no language or other restrictions. Additionally, we manually searched for relevant review articles and checked the bibliographic reference lists of studies selected for inclusion in our meta-analysis.

We included studies, discussing adult patients ***(P)*** with AP of different etiologies. We compared patients with FLD or NAFLD ***(E)*** to those without FLD or NAFLD ***(C).*** The eligible studies were supposed to define FLD or NAFLD based on abdominal imaging (ultrasound—US, computed tomography—CT scan, magnetic resonance imaging—MRI) or liver biopsy. In NAFLD the amount of alcohol consumed should also be defined. The primary outcome ***(O)*** was in-hospital mortality, secondary outcomes included AP severity [4], local complications (acute peripancreatic fluid collection—APFC, acute necrotic collection—ANC, pancreatic pseudocyst—PP), systemic inflammatory response syndrome (SIRS), and the length of hospitalization (LOH). We narrowed the focus to longitudinal studies.

### 2.2. Study Selection and Data Collection

We followed the recommendation of the Cochrane Handbook [21]. Two independent investigators (S.V., S.Z.) selected the studies, using EndNote X7.4 (Clarivate Analytics, Philadelphia, PA, USA). After removing duplicates, publications were screened for title and abstract. Two reviewers (S.V., S.Z.) assessed the studies meeting the eligibility criteria (PECO) for full-text. Conference abstracts reporting relevant data were also included. Disagreements were resolved by third party arbitration (P.H.).

The most recent publication was chosen in the case of multiple publications on the same cohort of patients.

Data were extracted independently by two investigators (S.V., Z.S.) into a pre-defined Excel datasheet (Office 365, Microsoft, Redmond, WA, USA). The following data were collected: first author, year of publication, study period, study design, demographic data, sample sizes, mean age, female percentage, details on the PECO question and data necessary for risk of bias assessment. For statistical analysis, we extracted raw data into 2 by 2 tables (outcome yes/no, FLD or NAFLD yes/no) and odds ratios (OR) for each outcome.

Graphical data were also extracted using GetData Graph Digitizer 2.26 software (S. Fedorov 2013, Russia, http://getdata-graph-digitizer.com).

### 2.3. Statistical Analysis

Meta-analytical calculations were performed in Stata 15.1 data analysis and statistical software (Stata Corp LLC, College Station, TX, USA) and Comprehensive Meta-Analysis (version 3, Biostat Inc., Englewood, NJ, USA) by a statistician (D.N.). For FLD vs. no-FLD and NAFLD vs no-NAFLD comparisons, we calculated pooled OR with 95% confidence interval (CI) with the random-effects model using the DerSimonian–Laird method [22] for in-hospital mortality, severity of AP, risk of local complications (ANC, APFC, PP) and SIRS, and weighted mean difference (WMD) with 95%CI for LOH.

Heterogeneity was tested by using the Cochrane’s Q and the I^2^ statistics, where I^2^ = 100% × (Q − df)/Q, and represents the magnitude of the heterogeneity (moderate: 30–60%, substantial: 50–90%, considerable: 75–100%). A p-value of less than 0.10 was considered suggestive of significant heterogeneity [23].

We performed sensitivity analysis (leave-one-out method) if at least three studies were included in an analysis by testing the effect of each study on the main association.

To test the presence of small-study effect we assessed the symmetry of the funnel plot visually.

### 2.4. Risk of Bias and Quality Assessment of the Individual Studies

A critical appraisal tool for prognostic studies, the Quality in Prognosis Studies (QUIPS) tool was used to assess the methodological quality of the included studies [24]. Two independent investigators (S.V., Z.S.) assessed the risk of bias; disagreements were resolved by discussion or by a third investigator. The main domain “study attrition” and further items not fitting our meta-analysis were omitted due to the retrospective design of the included studies.

### 2.5. Details of Ethical Approval

No ethical approval was required for this review as all data were already published in peer reviewed journals. No patients were involved in the design, conduct or interpretation of our review.

## 3. Results

### 3.1. Search and Selection

**Figure 1:**
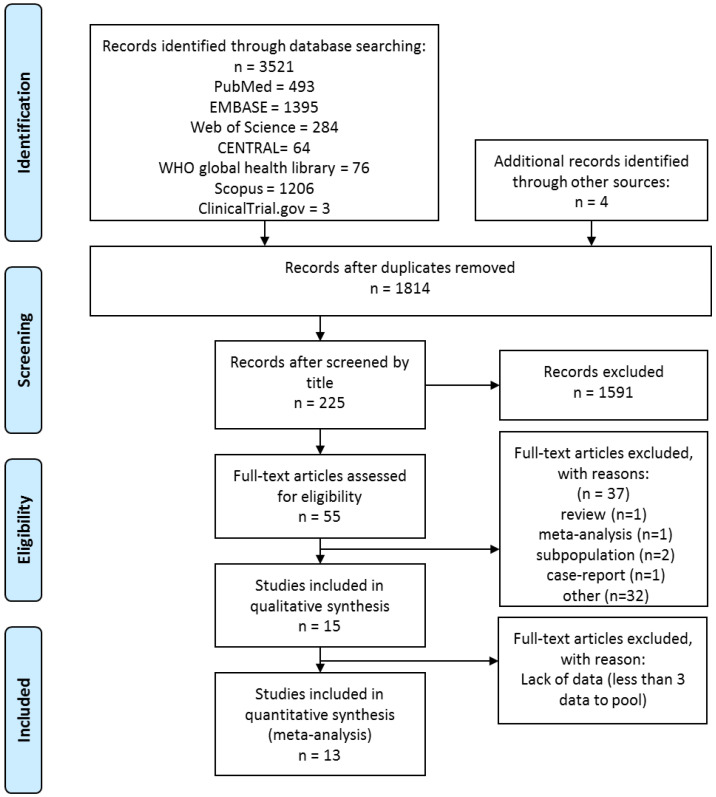
Preferred Reporting Items for Systematic Reviews and Meta-Analysis (PRISMA) flowchart for the study selection procedure.

Altogether 15 articles were eligible to be included in the systematic review, 13 of which in the meta-analysis. The details of the literature search are included in Figure 1. On full-text assessment we excluded six studies due to inappropriate study design; details are presented in Appendix S1.

### 3.2. Characteristics of the Studies Included in the Meta-Analysis

The main characteristics of the included studies are summarized in Table 1. All studies were retrospective cohort studies.

The Revised Atlanta Classification [4] and the Atlanta Classification of 1992 [25] were used in 11 of the included articles; furthermore, CTSI and magnetic resonance severity index—MRSI [10] were also used for AP severity classification.

The prevalence of FLD and NAFLD ranged from 18 to 82%, and from 24 to 58%, respectively. FLD and NAFLD was diagnosed using an unenhanced abdominal CT scan in 6 of 13 articles. Other studies used abdominal US or MRI to diagnose FLD or NAFLD, 2 out of 13 articles did not report the used method. Eligibility criteria from the studies included are summarized in Table S2.

### 3.3. Findings of Meta-Analysis: FLD vs. No FLD

Our findings are summarized in Table 2.

**Figure 2:**
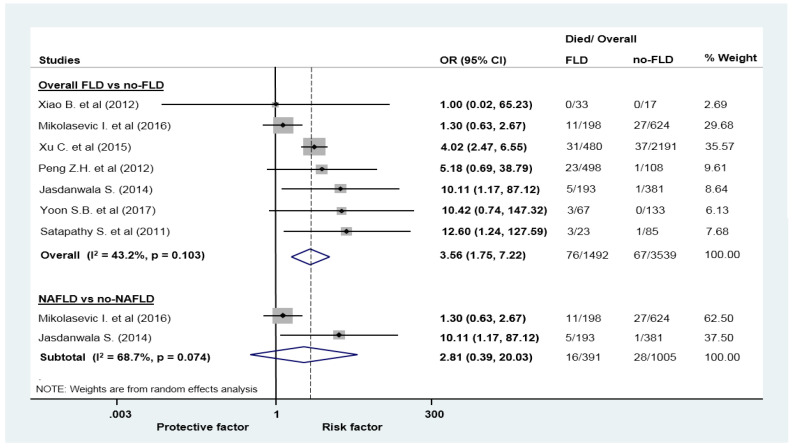
Forest plots of studies evaluating the association between fatty liver disease or non-alcoholic fatty liver disease and overall survival of patients with acute pancreatitis; CI: confidence interval, OR: odds ratio.

**Figure 3:**
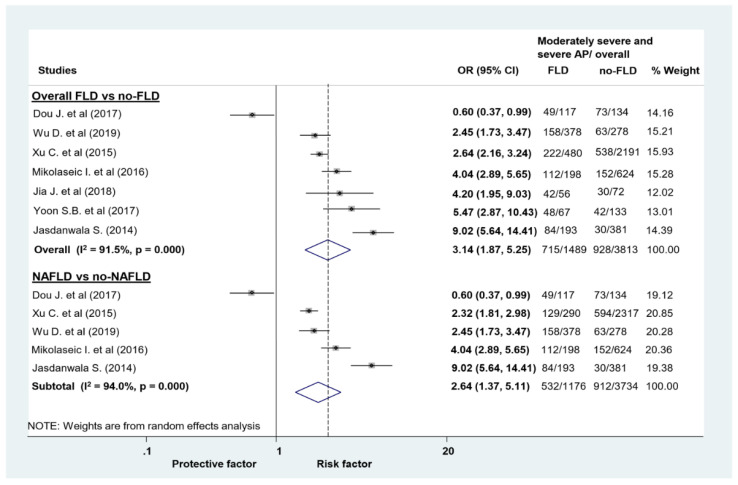
Forest plots of studies evaluating the association between fatty liver disease (FLD) or non-alcoholic fatty liver disease (NAFLD) and disease severity of acute pancreatitis (AP). We compared the odds of moderately severe/severe vs mild AP in patients with and without FLD/NAFLD; CI: confidence interval, OR: odds ratio.

In patients with AP, the odds of in-hospital mortality (5.09 vs. 1.89%; OR = 3.56, CI: 1.77–8.28; Figure 2), composite of moderately severe and severe AP (48.02 vs. 24.34%; OR = 3.14, CI: 1.87–5.25; Figure 3), and the odds of severe AP alone (16.33 vs. 7.87%; OR = 2.67, CI: 2.01–3.56; Figure S1) was higher in the FLD group compared with those without FLD.

In the subgroup of studies using the Atlanta 1992 classification for AP classification, in the FLD group the odds of severe AP was significantly higher (OR = 4.70, CI: 2.65–8.32; Figure S2).

**Figure 4:**
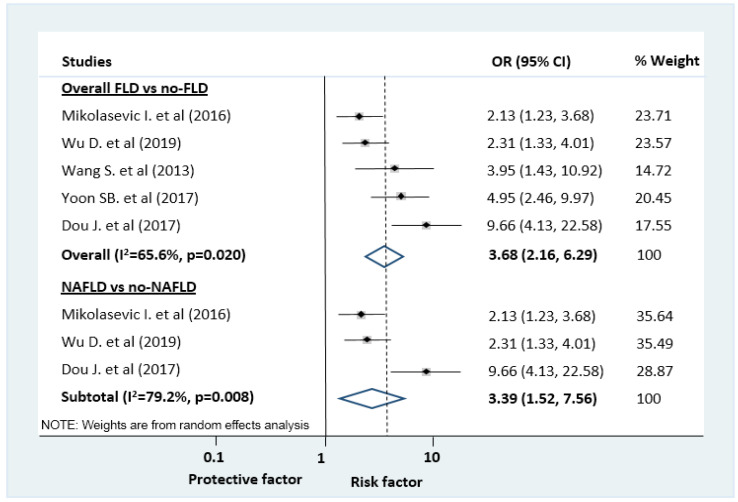
Forest plots of studies evaluating the association between fatty liver disease (FLD) or non-alcoholic fatty liver disease (NAFLD) and disease severity of acute pancreatitis (AP). Logistic regression analysis results were pooled, comparing the odds of moderately severe/severe vs mild AP in patients with and without FLD/ NAFLD; CI: confidence interval, OR: odds ratio.

In multivariate analysis (Figure 4), there was an independent association between FLD and the odds of moderately severe/ severe AP based on five studies (OR = 3.68, CI: 2.16–6.29). Details of the multivariate analysis adjustments in the included studies are summarized in Table S3.

**Figure 5:**
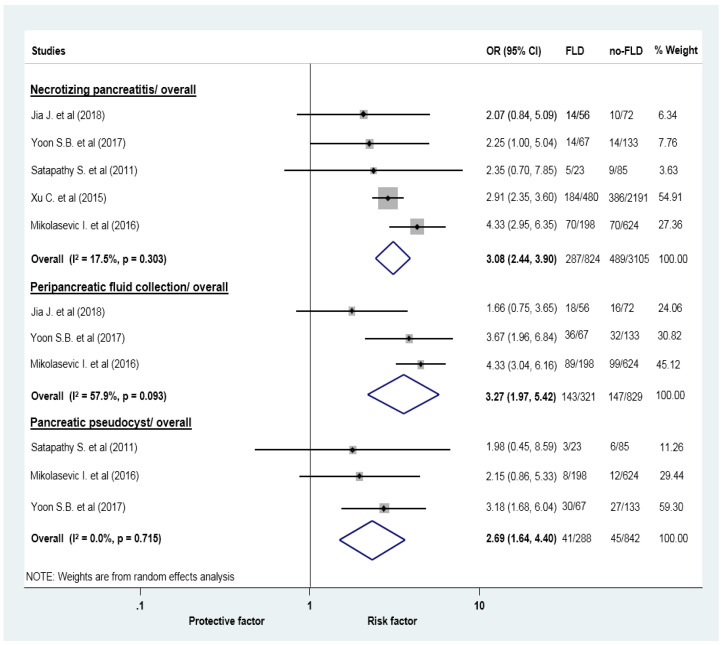
Forest plots of studies evaluating the association between fatty liver disease and the odds of local complications (necrotizing pancreatitis, peripancreatic fluid collection and pancreatic pseudocyst) in acute pancreatitis; CI: confidence interval, FLD: fatty liver disease, OR: odds ratio.

The proportion of acute necrotic collection (34.83 vs. 15.75%), acute peripancreatic collection (44.55 vs 17.73%), and peripancreatic pseudocyst (14.24 vs. 5.34) was higher in AP patients with FLD compared with the group without FLD (Figure 5). SIRS was also more frequent in AP patients with FLD (38.19 vs 18.63%; Figure S4).

Based on five articles, LOH was longer among patients with FLD than in the non-FLD patient group (WMD = 1.46 days, CI: 0.54–2.39 days; Figure S5).

The results of the heterogeneity analysis are presented in the figures corresponding to the assessed outcomes (Figure 2, Figure 3, Figure 4 and Figure 5; Figures S1–S5).

### 3.4. Findings of Meta-Analysis: NAFLD vs. No NAFLD

Although mortality in the NAFLD group was higher compared to those without it, the difference failed to attain the level of significance (OR = 2.81, CI: 0.39–20.03; Figure 2). Based on five articles, the course of AP was more severe in patients with NAFLD, the odds of moderately severe/severe AP was 2.64 higher (OR = 2.64, CI: 1.37–5.11; Figure 3). The odds to develop severe AP was also higher in the NAFLD group (OR = 2.21, CI: 1.70–2.88; Figure S3).

Based on 3 articles, NAFLD was an independent predictor of severe AP (OR = 3.39, CI: 1.52–7.56; Figure 4).

Patients with NAFLD tended to have longer hospital stay (WMD = 1.41 days, CI: 0.03–2.79 days; Figure S5).

### 3.5. Additional Analysis

The risk of bias and quality assessment of the individual studies are summarized in Table S4. Details of the risk of bias assessment are included in Appendix S1.

Funnel plots can be found in Figures S6, S7 and S8. According to the results, we did not observe evidence of publication bias when assessing funnel plots visually.

Sensitivity analysis, except for one outcome, showed no significant difference. When we removed the study of Yoon et al. [37] from the forest plot with the odds of pancreatic pseudocyst, the results became non-significant (OR = 2.09; CI: 0.97–4.55).

## 4. Discussion

As we know, this is the first meta-analysis to analyze the risk of multiple outcomes in AP patients with NAFLD.

Previously, only one meta-analysis that included a limited number of articles reported increased AP severity in FLD patients [39]. In this analysis, they reported on the severity of AP in patients with and without FLD, even though one of the included articles in their analysis reported on the association between severe FLD and AP severity. They did not manage to make a difference between FLD etiologies (alcoholic, non-alcoholic, metabolic etc.), even though it could have an impact on AP severity.

FLD is known to be associated with increased cardiovascular mortality and elevated risk of chronic kidney disease [40]. Fatty liver is common in AP patients because both conditions share contributing factors such as obesity, alcohol abuse, or hyperlipidemia, but its association with the prognosis of AP is still unclear.

Based on pooled data, AP patients with FLD were more likely to die during in-hospital stay than those in the non-FLD group. Eight of the included articles in this meta-analysis found a clear association between FLD and the development of severe AP. The rate of moderately severe/severe AP was also higher in AP patients with NAFLD, with significantly longer in-hospital stay, however the rate of mortality did not reach a significant difference. Overall, AP patients with FLD and NAFLD had a more severe disease course, an increased risk for the development of both local and systemic complications, and also a longer in-hospital stay.

Guidelines recommend performing a contrast-enhanced CT scan within 72 h–96 h after the onset of the AP symptoms [6]. Combined unenhanced and enhanced CT scans may be useful in assessing the status of both AP and FLD [37]. Studies that used CT scan and US or other methods (US elastography, MRI, etc.) have all shown acceptable levels of sensitivity for detecting FLD [3,41]. According to international guidelines, US should be used on the first hand to diagnose FLD since it is more widely available and cheaper than the gold standard MRI. However, US has limited specificity and does not reliably detect steatosis when <20%, compared to the MRI that can detect 5% fat in the liver. Another clinically available imaging technique, the controlled attenuation parameter (CAP) can diagnose FLD which classifies the steatosis in three grades based on the amount of liver with fatty change [3].

Significant heterogeneity could be observed among the cause of AP and FLD. According to Yoon et al. [37], a strong trend between the presence of FLD and AP severity was observed regardless of the cause of pancreatitis (alcoholic vs. non-alcoholic). Xu et al. [18] have found no difference in AP severity when comparing alcoholic FLD with NAFLD. In both cases the course of AP was worse compared to non-FLD patients.

MetS is often seen in patients with FLD. According to Szentesi et al. [13], the presence of two, three, or four MetS factors significantly increased the rate of worse outcome parameters by 9.5, 24.1, and 66.7%, respectively. In this analysis, only hypertriglyceridemia was independently associated with a more severe course of AP (OR = 3.41, 95%CI: 1.39–8.37).

Based on four articles [18,19,32,42], the severity of FLD affects AP outcomes. All these findings imply that the severity of FLD has a negative impact on the course of AP. Wang et al. [43] also reported a higher rate of severe AP in patients with severe FLD. On the other hand, the course of AP was more severe in cirrhotic patients [44]; however, the higher rate of mortality was attributed to complications of cirrhosis.

Results regarding AP severity defined by score systems were also reported in five of the included studies. Significantly higher BISAP scores (mean BISAP 0.813 vs. 0.544, *p* < 0.01) [28] and in two articles significantly higher CTSI scores were reported in FLD patients compared to non-FLD patients (mean CTSI 2.9 vs. 1.1, *p* < 0.01 and 4 vs. 2.2, *p* < 0.05) [30,33]. APACHE-II score was also significantly higher (mean APACHE-II 8.4 vs. 7.2, *p* < 0.01) in one of the included studies [30].

Four of the included articles suggested the incorporation of FLD into prognostic tools, but only Hao et al. [27] analyzed the effect of inclusion of FLD in the APACHE-II score system. They reported increased sensitivity and specificity when predicting severe AP (78.1% vs 85.4% and 86.2% vs 75.5%).

While Ding et al. [45] reported a non-significant effect of FLD on pancreatic necrosis infection (OR = 0.971; 95% CI: 0.45–2.08), another study reported an increased risk of infection in AP patients with FLD (46.5% vs. 38%, *p* < 0.05) [18]. Satapathy et al. [33] reported an increased need for antibiotics in AP patients with FLD (69.6% vs. 30.6%). This data was only represented in a few articles and therefore was not suitable for quantitative analysis.

FLD was also associated with increased hospital readmission of patients with AP (OR = 3.48, 95% CI: 1.70–7.11). However, data were collected retrospectively and admission diagnosis of acute or chronic pancreatitis were screened together regarding later readmission with a pancreatitis-related diagnosis [34].

According to Yuan et al. [38], fatty liver was a risk factor for abnormal fasting blood glucose levels (HR = 1.869, 95% CI = 1.16–3.01) after the first episode of AP. The median follow-up period in the study was three years; however, the definition of FLD was not reported. None of the included studies in the analysis discussed long-term complications.

### Strengths and Limitations

Our meta-analysis has several strengths, most importantly, the rigorous methodology. We performed a systematic search followed by reproducible selection and data extraction. The strengths of this study also include the covariate-adjusted for AP severity and the high number of AP cases.

Several limitations should be taken into consideration when interpreting our conclusions. First, we included conference abstracts to reduce the risk of publication bias, but these are often lacking details; therefore, they are subjected to a potential risk of bias. Due to the low number of studies included (<10), we were unable to test if publication bias affects the results. All the included articles were retrospective, single-center cohort studies. Most of the study populations came from Asia, with a potential bias when making general conclusions, and may not be representative of other geographical regions. The diagnosis of AP and FLD was not uniformly used in the included articles. Neither of the included studies confirmed FLD in patients with liver biopsy. Not all the included articles reported the timing of repeated abdominal imaging; therefore, a potential heterogeneity is present in the rate of local complications. Significant heterogeneity could be found in some of the results (severity, independent risk, and peripancreatic fluid collection). Sensitivity analysis showed significant difference just in the case of one outcome (the odds of pancreatic pseudocyst).

Risk factors included in the individual logistic regression analysis were not uniform between the studies (Appendix S1, Table S3).

## 5. Conclusions

### 5.1. Implication for Practice

Our results showed that FLD and NAFLD worsen the course of AP. FLD and NAFLD can be easily diagnosed by abdominal US (affordable, non-invasive investigation) or abdominal CT scan (high sensitivity and specificity). We suggest that, compared to the current practice, a different approach should be taken into consideration in AP patients, and an initial non-invasive assessment of not only the pancreas but also the liver to detect fatty liver may be beneficial for patients with AP and may help to consider more individualized patient care.

### 5.2. Implication for Research

Since FLD and NAFLD may have an essential impact on AP outcomes, we suggest the incorporation of the assessment of FLD and NAFLD into the prognostic tools applied in the case of AP. Long-term complications were not assessed in the included studies; follow-up results are needed. AP associated with FLD may result in higher health care utilization and costs of medical services. The detailed economic impact of the FLD and NAFLD should be analyzed in patients with AP. Possible treatment options to decrease the increased risks of AP complications should be researched.

## Figures and Tables

**Table 1:** Characteristics of the studies included in the systematic review and meta-analysis.

Author and Year	Country (Centre)	Recruitment Period	Acute Pancreatitis Diagnosis	Leading Etiology of Acute Pancreatitis	Nr. of Acute Pancreatitis Cases	Fatty Liver Disease			Examined Outcomes
						Definition	Diagnostic Method (Cut-Off)	Nr. FLD Cases (%)	
Dou J. et al., 2017 [26] (article in Chinese)	China (single-center)	2013–2016	2 out of 3 criteria	G 37% H 10%	251	NAFLD	US (NR)	117 (47)	AP severity (Atlanta 2012) ^§^
Hao Y.M. et al., 2015 [27] ^†^	China (single-center)	2011–2013	NR	NR	148	FLD	NR	41 (28)	AP severity (Atlanta 1992)
Jasdanwala S, 2015 [28]	USA (multicenter)	Not reported	2 out of 3 criteria	NR	574	NAFLD	CT or US (NR)	193 (34)	In-hospital mortality, AP severity (Atlanta 2012), LOH, ICU admission, BISAP
Jia J. et al., 2018 [29]	China (single-center)	2016–2017	2 out of 3 criteria	NR	128	FLD	CT (HAI<1)	56 (44)	AP severity (Atlanta 2012), ANC, APFC
Mikolasevic I. et al., 2016 [30]	Croatia (single-center)	2008–2015	2 out of 3 criteria	G 84% H 1%	822	NAFLD	CT (HA > 10 HU, or LD < 40 HU) or US	198 (24)	In-hospital mortality, AP severity (Atlanta 2012) ^§^, ANC, APFC, PP, LOH, APACHE-II, CTSI
Morel C.E. et al., 2019 [31] (article in Spanish)	Mexico (single-center)	2017–2018	2 out of 3 criteria	G 70% A 11% H 5%	186	FLD	US (NR)	68 (37)	AP severity (Atlanta 2012), persistent SIRS
Peng Z.H. et al., 2012 [32] (article in Chinese)	China (single-center)	2010–2011	2 out of 3 criteria	G 57%	606	FLD	CT (HAI < 1)	498 (82)	In-hospital mortality, overall complications ^§^
Satapathy S. et al., 2011 [33] ^†^	USA (single-center)	2002–2009	NR	G 39% A 18%	108	FLD	CT (HAI < 0.8)	23 (21)	In-hospital mortality, ANC, PP, LOH, ICU admission, need for antibiotics, CTSI, Ranson 48 h
Suchsland T. et al., 2015 [34]	Germany (single-center)	2006–2011	ICD-10	NR	373	FLD	NR	NR	Risk of hyperglycemia after AP
Wang S. et al., 2013 [35] ^†^	China (single-center)	2010–2011	NR	NR	120	FLD	NR	35 (29)	AP severity (Atlanta 1992) ^§^, SIRS, pulmonary failure, metabolic disturbances
Wu D. et al., 2019 [19]	China (single-center)	2012–2016	2 out of 3 criteria	G 32% H 48%	656	NAFLD	CT (HAI < 1)	378 (58)	AP severity (Atlanta 2012) ^§^, SIRS, BISAP, Ranson score
Xiao B. et al., 2012 [36]	China (single-center)	2009–2011	Pain and laboratory results ^‡^	G 38%	50	FLD	MRI (HAI)	33 (66)	In-hospital mortality, MRSI
Xu C. et al., 2015 [18]	China (single-center)	2000–2014	2 out of 3 criteria	G 58% A 22% H 11%	2671	FLD/ NAFLD	CT (HAI < 1)	480 (18)	In-hospital mortality, AP severity (Atlanta 2012), ANC, systemic and local complications, APACHE-II
Yoon S.B. et al., 2017 [37]	Korea (single-center)	2009–2016	2 out of 3 criteria	G 36% A 34% H 3%	200	FLD	CT (HAI < 1)	67 (34)	In-hospital mortality, AP severity (Atlanta 2012) ^§^, ANC, PP, APFC, LOH
Yuan L. et al., 2017 [38]	China (single-center)	2009–2013	2 out of 3 criteria	G 49% A 5% H 10%	310	FLD	NR	119 (39)	hospital readmission after the first episode of AP

^†^: conference abstract; ^‡^: AP diagnostic criteria were based on abdominal pain and serum pancreatic enzyme elevation; ^§^: outcome assessed by adjusted analysis from logistic regression; 2 out of 3 criteria: 1. abdominal pain, 2. laboratory findings, 3. abdominal imaging [4]; AFLD: alcoholic fatty liver disease; ANC: acute necrotic collection; AP: acute pancreatitis; APACHE-II: “Acute Physiology, Age, Chronic Health Evaluation II”; APFC: acute peripancreatic fluid collection; BISAP: bedside index for severity in acute pancreatitis; CT: computed tomography; CTSI: CT severity index; Etiology A: alcohol abuse, G: gallstone disease, H: hypertriglyceridemia induced; ICU: intensive-care unit; ICD-10: International Statistical Classification of Diseases and Related Health Problems 10th revision; FLD: fatty liver disease; HA: hepatic attenuation; HAI: hepatic attenuation index; LD: liver density; LOH: length of hospitalization; MRI: magnetic resonance imaging; MRSI: magnetic resonance severity index; NAFLD: non-alcoholic fatty liver disease; PP: pancreatic pseudocyst; SIRS: systemic inflammatory response syndrome; US: abdominal ultrasound; USA: United States of America.

**Table 2:** Summary of findings.

Outcome	N0 of Studies (N0 of PTS)	Odds Ratio (95% CI)	I^2^ (%)	Chi^2^
**FLD vs no-FLD**				
Mortality	7 (5031)	3.56 (1.77–8.28)	43.2	0.103
Composite of MSAP and SAP (uni)	7 (5302)	3.14 (1.87–5.25)	91.5	0
Composite of MSAP and SAP (multi) ^‡^	5 (NR)	3.68 (2.16–6.29)	65.6	0.020
SAP by Atlanta 2012	8 (4931)	2.67 (2.01–3.56)	32.0	0.173
SAP by Atlanta 1992	2 (268)	4.70 (2.65–8.32)	0	0.634
Acute necrotic collection	5 (3929)	3.08 (2.44–3.90)	17.5	0.303
Acute peripancreatic fluid collection	3 (1150)	3.27 (1.97–5.42)	57.9	0.093
Pancreatic pseudocyst	3 (1130)	2.69 (1.64–4.40)	0	0.715
SIRS	4 (3634)	2.39 (1.74–3.28)	47	0.129
Length of hospital stay	5 (1955)	1.46 (0.54–2.39) †	40.7	0.150
**NAFLD vs no-NAFLD**				
Mortality	2 (1396)	2.81 (0.39–20.03)	68.7	0.074
Composite of MSAP and SAP (uni)	5 (4910)	2.64 (1.37–5.11)	94	0
Composite of MSAP and SAP (multi) ^‡^	3 (NR)	3.39 (1.52–7.56)	79.2	0.008
SAP by Atlanta 2012	3 (4085)	2.21 (1.70–2.88)	0	0.806
Length of hospital stay	3 (1647)	1.41 (0.03–2.7) ^†^	68.5	0.042

CI = confidence interval, FLD = fatty liver disease, I2 and Chi2 = heterogeneity, MSAP = moderately severe acute pancreatitis, NAFLD = non-alcoholic fatty liver disease, SAP = severe acute pancreatitis, SIRS = systemic inflammatory response syndrome; ^†^ Length of hospital stay results are represented as weighted mean differences with 95% CI, values represent days; ^‡^ parameters included in multivariate analyses in the included studies are summarized in Table S3.

## Supplementary Materials

The following are available online at https://www.mdpi.com/2077-0383/9/9/2698/s1, **Table S1**: PRISMA checklist, **Table S2**: Inclusion and/or exclusion criteria in each included study in the systematic review and meta-analysis, **Table S3**: Factors included in multivariate logistic regression analyses, **Table S4:** Risk of bias assessment using QUIPS (Quality In Prognosis Studies) tool; **Figure S1**: Odds ratio of severe AP vs mild and moderately severe AP, comparing patients with FLD vs no-FLD, **Figure S2**: Odds ratio of severe AP vs mild AP, comparing patients with FLD vs no-FLD. Acute pancreatitis severity was defined based on the Atlanta Classification (1992) into mild and severe AP, **Figure S3**: Odds ratio of severe AP vs mild and moderately severe AP, comparing patients with NAFLD vs no-NAFLD, **Figure S4**: Forest plot representing the odds of SIRS in FLD and no-FLD patients suffering from AP. SIRS was defined as 2 or more of the included criteria, **Figure S5**: Forest plot representing the differences in length of hospitalization in FLD and no-FLD patients suffering from AP. Subgroup analysis with AP patients with NAFLD was also represented graphically. Data is described as number of patients included in the analysis (n) and mean hospital stay with standard deviation (SD), **Figure S6**: Funnel plot with pseudo 95% confidence intervals with included studies on Figure 2, **Figure S7**: Funnel plot with pseudo 95% confidence intervals with included studies on Figure 3, **Figure S8**: Funnel plot with pseudo 95% confidence intervals with included studies on Figure S1; **Appendix S1**: Results, Risk of bias assessment between studies.
