# Supplementary material for: Fatty Liver Disease and Non-Alcoholic Fatty Liver Disease Worsen the Outcome in Acute Pancreatitis: A Systematic Review and Meta-Analysis
Source: J Clin Med. 2020 Aug 20;9(9):2698. doi: 10.3390/jcm9092698 (PMC7564684; doi:10.3390/jcm9092698)
Supplement: Supplementary file 1 [file jcm-09-02698-s001.pdf]

Table S1. PRISMA checklist [1].

Section/topic	#	Checklist item	Reported on page #
**TITLE**			
Title	1	Identify the report as a systematic review, meta-analysis, or both.	1
**ABSTRACT**			
Structured summary	2	Provide a structured summary including, as applicable: background; objectives; data sources; study eligibility criteria, participants, and interventions; study appraisal and synthesis methods; results; limitations; conclusions and implications of key findings; systematic review registration number.	1
**INTRODUCTION**			
Rationale	3	Describe the rationale for the review in the context of what is already known.	1-2
Objectives	4	Provide an explicit statement of questions being addressed with reference to participants, interventions, comparisons, outcomes, and study design (PICOS).	2
**METHODS**			
Protocol and registration	5	Indicate if a review protocol exists, if and where it can be accessed (e.g., Web address), and, if available, provide registration information including registration number.	2
Eligibility criteria	6	Specify study characteristics (e.g., PICOS, length of follow-up) and report characteristics (e.g., years considered, language, publication status) used as criteria for eligibility, giving rationale.	2
Information sources	7	Describe all information sources (e.g., databases with dates of coverage, contact with study authors to identify additional studies) in the search and date last searched.	2
Search	8	Present full electronic search strategy for at least one database, including any limits used, such that it could be repeated.	2
Study selection	9	State the process for selecting studies (i.e., screening, eligibility, included in systematic review, and, if applicable, included in the meta-analysis).	2-3
Data collection process	10	Describe method of data extraction from reports (e.g., piloted forms, independently, in duplicate) and any processes for obtaining and confirming data from investigators.	3
Data items	11	List and define all variables for which data were sought (e.g., PICOS, funding sources) and any assumptions and simplifications made.	3
Risk of bias in individual studies	12	Describe methods used for assessing risk of bias of individual studies (including specification of whether this was done at the study or outcome level), and how this information is to be used in any data synthesis.	3
Summary measures	13	State the principal summary measures (e.g., risk ratio, difference in means).	3
Synthesis of results	14	Describe the methods of handling data and combining results of studies, if done, including measures of consistency (e.g., I^2) for each meta-analysis.	3
Risk of bias across studies	15	Specify any assessment of risk of bias that may affect the cumulative evidence (e.g., publication bias, selective reporting within studies).	3

Section/topic	#	Checklist item	Reported on page #
Additional analyses	16	Describe methods of additional analyses (e.g., sensitivity or subgroup analyses, meta-regression), if done, indicating which were pre-specified.	3
**RESULTS**			
Study selection	17	Give numbers of studies screened, assessed for eligibility, and included in the review, with reasons for exclusions at each stage, ideally with a flow diagram.	3
Study characteristics	18	For each study, present characteristics for which data were extracted (e.g., study size, PICOS, follow-up period) and provide the citations.	4
Risk of bias within studies	19	Present data on risk of bias of each study and, if available, any outcome level assessment (see item 12).	9-10
Results of individual studies	20	For all outcomes considered (benefits or harms), present, for each study: (a) simple summary data for each intervention group (b) effect estimates and confidence intervals, ideally with a forest plot.	9-10
Synthesis of results	21	Present results of each meta-analysis done, including confidence intervals and measures of consistency.	7-8
Risk of bias across studies	22	Present results of any assessment of risk of bias across studies (see Item 15).	9-10
Additional analysis	23	Give results of additional analyses, if done (e.g., sensitivity or subgroup analyses, meta-regression [see Item 16]).	9-10
**DISCUSSION**			
Summary of evidence	24	Summarize the main findings including the strength of evidence for each main outcome; consider their relevance to key groups (e.g., healthcare providers, users, and policy makers).	10
Limitations	25	Discuss limitations at study and outcome level (e.g., risk of bias), and at review-level (e.g., incomplete retrieval of identified research, reporting bias).	11
Conclusions	26	Provide a general interpretation of the results in the context of other evidence, and implications for future research.	11-12
**FUNDING**			
Funding	27	Describe sources of funding for the systematic review and other support (e.g., supply of data); role of funders for the systematic review.	13

Table S2. Inclusion and/or exclusion criteria in each included study in the systematic review and meta-analysis.

Study	Acute pancreatitis eligibility („verbatim")	Fatty liver disease eligibility („verbatim")
Dou J. et al., 2017 [2]	**Exclusion:** "(1) previous history of pancreatic disease, including acute pancreatitis, chronic pancreatitis, pancreatic cancer; (2) those with chronic heart disease; (3) those with chronic renal failure; (4) with chronic liver Those with dysfunction; (5) those with a history of malignancy; (6) those with a history of diabetes; (7) those with missing or incomplete data."	"Guidelines for the Diagnosis and Treatment of Nonalcoholic Fatty Liver Diseases 2010"
Hao Y.M. et al, 2015 [3]	Not reported	Not reported
Jasdanwala S, 2015 [4]	Not reported	"Significant alcohol consumption: more than 21 drinks per week in men and more than 14 drinks per week for women over a minimum 2 years period"
Jia J. et al, 2018 [5]	**Inclusion:** "diagnosis based on the two out of three criteria;" **Exclusion: "**a) no abdominal CT scan was performed within 24 hours; b) incomplete clinical data; c) Splenectomy patients."	Not reported
Mikolasevic I. et al, 2016 [6]	**Exclusion: "**relapse of acute pancreatitis or with an exacerbation of chronic pancreatitis, patients with incomplete medical data, patients with active malignancy, those who were younger than 18 years and those who were receiving medications that can cause liver steatosis (corticosteroids, amiodarone, etc.) **unknown etiology"**	**Exclusion: "**other causes of chronic steatosis; consummation of more than 14 alcohol drinks/week in women and more than 21 alcohol drinks/week in men was considered as excessive alcohol consumption; laboratory results indicating on possible alcohol consumption"
Morel C.E. et al, 2019 [7]	**Exclusion: "**chronic hepatitis, chronic pancreatitis, pancreatic cancer, incomplete data"	Not reported
Peng Z.H. et al, 2012 [8]	**Exclusion: "**CT not performed, abdominal surgery, decompensated cirrhosis, hypoproteinaemia (<30g/l), heart failure, infectious disease, malignancy, bleeding disorder"	Not reported
Satapathy S. et al, 2011 [9]	Not reported	Not reported
Suchsland T. et al, 2015 [10]	**Inclusion:** "patients that were treated at University Medicine Greifswald with the main diagnosis acute pancreatitis (ICD-10-GM: K85.xx) or chronic pancreatitis ICD-10-GM: K86.0 (alcoholic chronic pancreatitis) or K.86.1 (chronic pancreatitis by other origin) between 2006 and 2011." **Exclusion:** "Patients with incomplete or inconsistent information from the HIS were excluded. When data from the questionnaire were incomplete, the existing information was still analyzed in bivariate analyses but could not include in multivariate analyses because of the test design."	Not reported

Study	Acute pancreatitis eligibility („verbatim”)	Fatty liver disease eligibility („verbatim”)
Wang S. et al, 2013 [11]	Not reported	Not reported
Wu D. et al, 2019 [12]	**Exclusion:** "patients suffering from cirrhosis, hepatocellular carcinoma, alcoholic fatty liver, or chronic pancreatitis as well as those who had undergone splenectomy, were pregnant, were younger than 18 or older than 60 years, had been hospitalized repeatedly, or had incomplete medical"	**Exclusion: "**history of alcoholic consumption (history of drinking or equivalent alcohol consumption of more than 140 g/week for men and more than 70 g/week for women), viral hepatitis, drug-induced hepatitis, total parenteral nutrition, hepatolenticular degeneration, autoimmune liver disease, and other specific diseases that can lead to fatty liver"
Xiao B. et al, 2012 [13]	**Exclusion: "**patients with a history of diabetes mellitus, obesity (body mass index Q28 kg/m2), alcohol consumption (960 g/d for 91 year), type B/type C viral hepatitis, hepatic cirrhosis, or cancer proved by clinical, imaging, or histological evidence"	**Inclusion: "**MRI performed within 72 hours after the onset of symptoms, MRI was followed by collection of blood samples, 1 or more MRI follow-ups, including a review of the results"
Xu C. et al, 2015 [14]	**Exclusion: "**chronic cardiac and pulmonary diseases; previous history of pancreatic diseases, including acute pancreatitis, chronic pancreatitis and pancreatic cancer; chronic renal failure; chronic liver dysfunction; a history of malignancy."	**"Non-alcoholic fatty liver disease (NAFLD)** was diagnosed by the presence of following findings: (1) steatosis was detected either by imaging or histology; (2) the alcoholic liver disease was excluded, and alcohol consumption was less than 140g per week in men (70g in women) in the past 12 months; (3) specific diseases that could lead to steatosis were excluded as mentioned above"
Yoon S.B. et al, 2017 [15]	**Exclusion: "**ERCP, reffered cases from other hospitals without u CT, missing BMI data"	**Exclusion: "**referred cases from other hospitals without an initial CT study, cases without CT scan or unenhanced CT phase"
Yuan L. et al, 2017 [16]	**Inclused: "**contact telephone number and met the diagnostic criteria of a first attack of AP were included in the study.<br>Patients who fulfilled 1 or more of the following criteria were **excluded:** (1) previous diagnosis of DM, impaired fasting glucose (IFG), or impaired glucose tolerance; (2) abnormal glycosylated hemoglobin (HbA1c) during the course of hospitalization; (3) previous AP attack before the beginning of the cohort and history of other pancreatic injury, including chronic, autoimmune, or hereditary pancreatitis, trauma, treatment of pancreatectomy or debridement, pancreatic neoplasm, cystic fibrosis, hemochromatosis, or fibrocalculous pancreatopathy; (4) previous history of hyperthyroidism, decompensated cirrhosis, ormalignant neoplasm; (5) lack of regular monitoring of FBG before or after AP; (6) history of gestational DM; (7) death during hospitalization; and (8) loss to follow-up."	Not reported

Table S3. – Factors included in multivariate logistic regression analyses.

**Studies**	**Factors included in the multivariate analysis**
Yoon S.B. et al, 2017 [15]	Age, gender, body mass index, alcohol consumption
Mikolasevic I. et al, 2016 [6]	Arterial hypertension, type 2 diabetes mellitus, dyslipidemia, body mass index
Wang S. et al, 2013 [11]	Age, gender, etiology, systemic complications, pulmonary failure
Wu D. et al, 2019 [12]	Age, gender, body mass index, serum triglyceride level, chronic heart disease, type 2 diabetes mellitus, arterial hypertension, smoking
Dou J. et al, 2017 [2]	Body mass index, white blood cells, serum amylase level

Table S4. Risk of bias assessment using QUIPS (Quality In Prognosis Studies) tool.

**Study**	**1[a]**	**2[b]**	**3**	**4**	**5**	**6**
Dou J. et al [2]	Green	N/ A	Green	Green	Green	Green
Hao Y.M. [3]	Red	N/ A	Red	Yellow	Red	Red
Jasdanwala S. [4]	Green	N/ A	Yellow	Green	Yellow	Green
Jia J. et al [5]	Green	N/ A	Green	Green	Yellow	Green
Mikolasevic I. et al [6]	Green	N/ A	Green	Green	Green	Green
Morel C.E. et al [17]	Green	N/ A	Yellow	Green	Yellow	Green
Peng Z.H. et al [8]	Green	N/ A	Green	Green	Yellow	Green
Satapathy S. et al [9]	Yellow	N/ A	Green	Yellow	Red	Red
Wang S. et al [11]	Yellow	N/ A	Red	Yellow	Yellow	Red
Wu D. et al [12]	Green	N/ A	Green	Green	Green	Green
Xiao B. et al [13]	Green	N/ A	Green	Green	Green	Green
Xu C. et al [14]	Green	N/ A	Green	Green	Green	Green
Yoon S.B. et al [15]	Green	N/ A	Green	Green	Green	Green

Items in columns 1: Study participation, 2: Study attrition, 3: Prognostic factor measurement, 4: Outcome measurement, 5: Study confounding, 6: Statistical analysis and reporting; **Green:** low risk of bias, **yellow:** moderate risk of bias, **red:** high risk of bias; **a.** Overall ratings for each domain was assigned as carrying 'low', 'moderate' or 'high' risk of bias, based on the items included in each domain; **b.** N/A: not applicable.

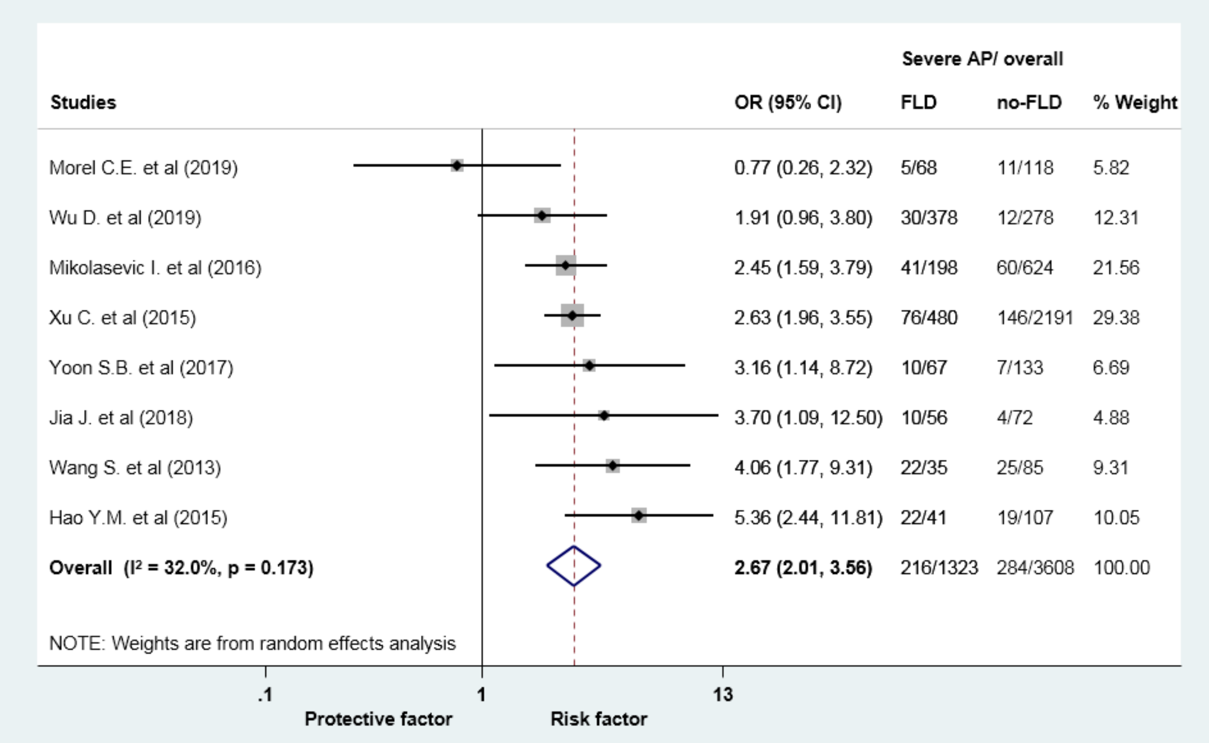

Figure S1. Odds ratio of severe AP vs mild and moderately severe AP, comparing patients with FLD vs no-FLD. AP: acute pancreatitis, CI: confidence interval, FLD: fatty liver disease, OR: odds ratio

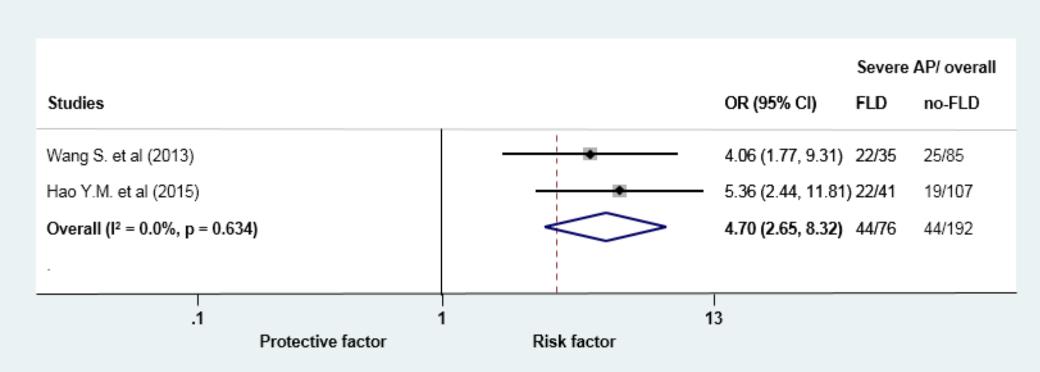

Figure S2. Odds ratio of severe AP vs mild AP, comparing patients with FLD vs no-FLD. Acute pancreatitis severity was defined based on the Atlanta Classification (1992) into mild and severe AP. AP: acute pancreatitis, CI: confidence interval, FLD: fatty liver disease, OR: odds ratio

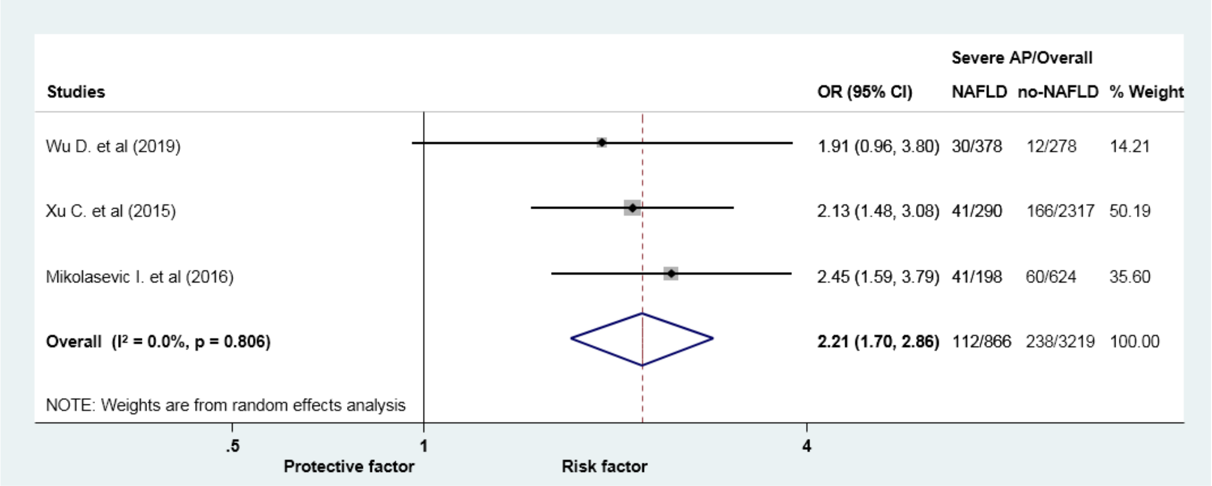

Figure S3. Odds ratio of severe AP vs mild and moderately severe AP, comparing patients with NAFLD vs no-NAFLD. AP: acute pancreatitis, CI: confidence interval, NAFLD: non-alcoholic fatty liver disease, OR: odds ratio

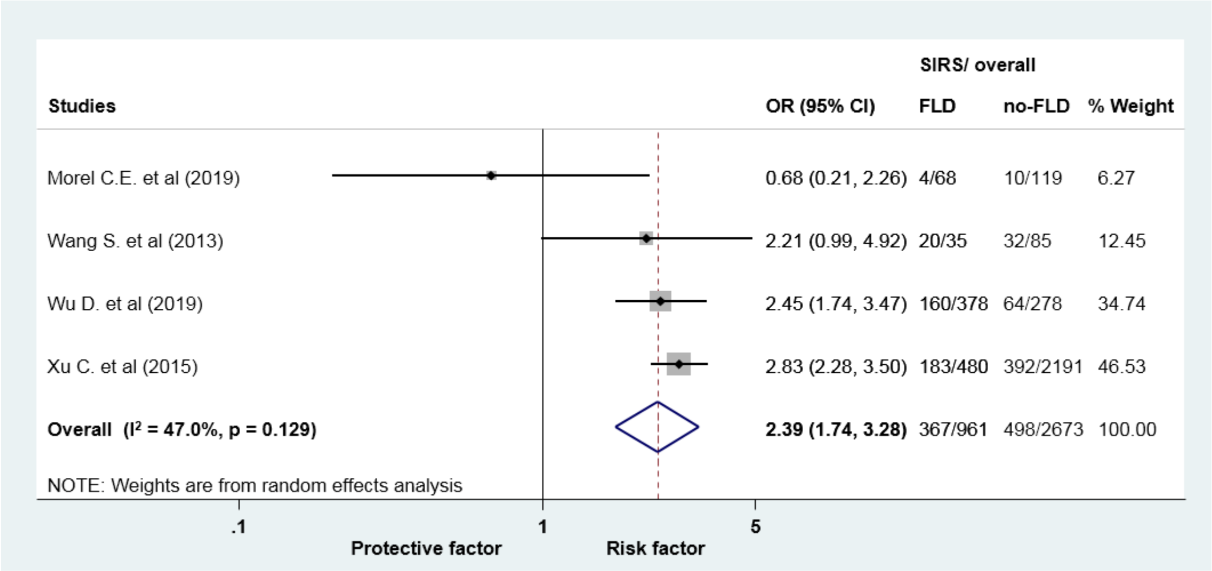

Figure S4. Forest plot representing the odds of SIRS in FLD and no-FLD patients suffering from AP. SIRS was defined as 2 or more of the included criteria.

AP: acute pancreatitis, CI: confidence interval, FLD: fatty liver disease, OR: odds ratio, SIRS: systemic inflammatory response syndrome

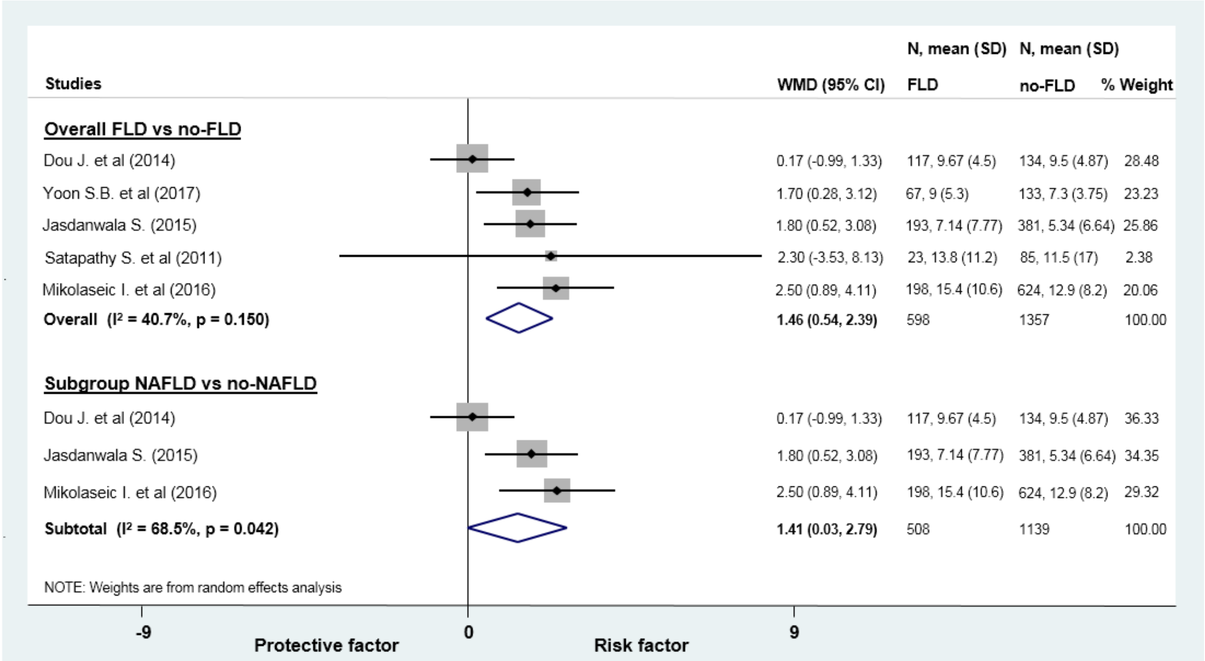

Figure S5. Forest plot representing the differences in length of hospitalization in FLD and no-FLD patients suffering from AP. Subgroup analysis with AP patients with NAFLD was also represented graphically. Data is described as number of patients included in the analysis (n) and mean hospital stay with standard deviation (SD). AP: acute pancreatitis, CI: confidence interval, FLD: fatty liver disease, N: number, NAFLD: non-alcoholic fatty liver disease, SD: standard deviation, WMD: weighted mean difference.

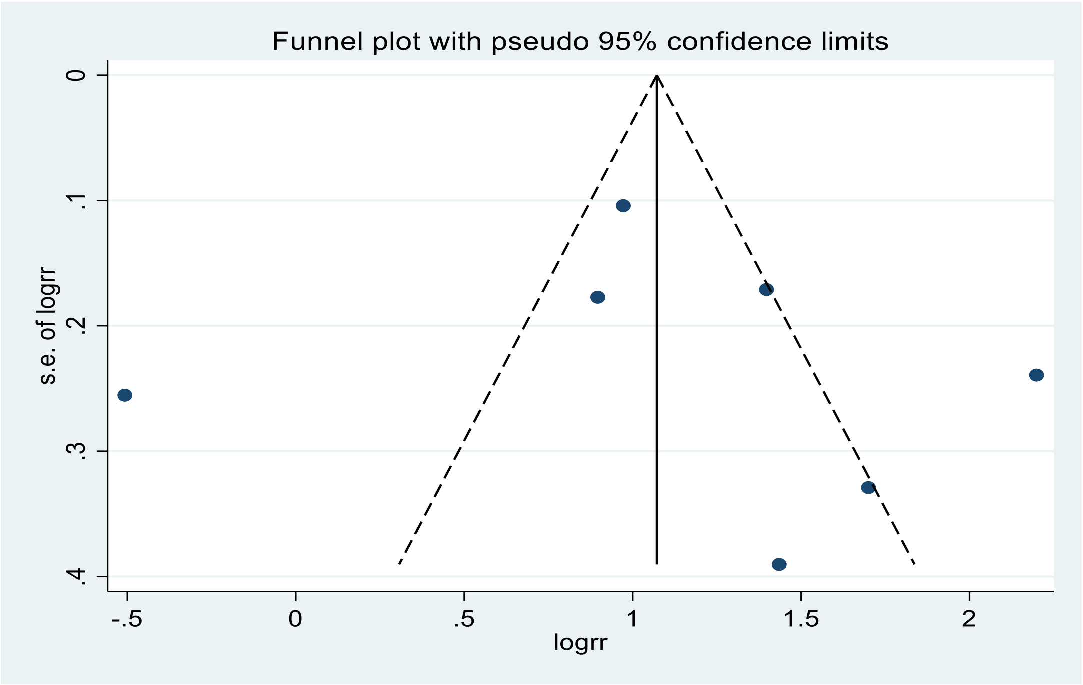

Figure S6. Funnel plot with pseudo 95% confidence intervals with included studies on Figure 2.

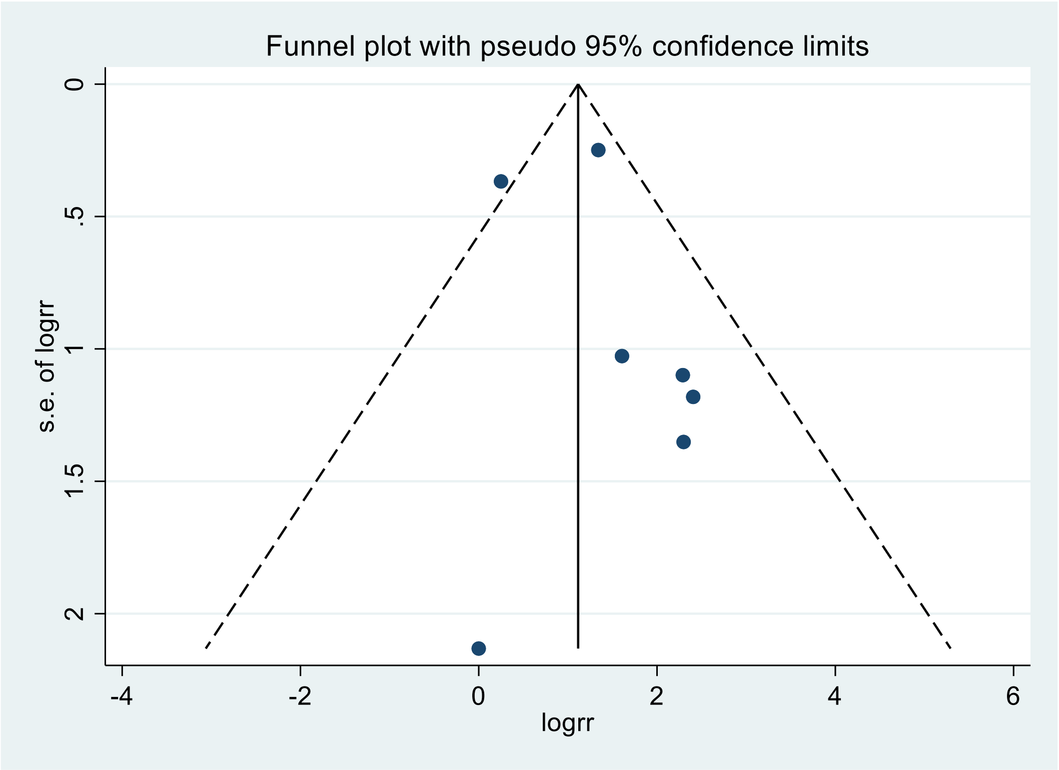

Figure S7. Funnel plot with pseudo 95% confidence intervals with included studies on Figure 3.

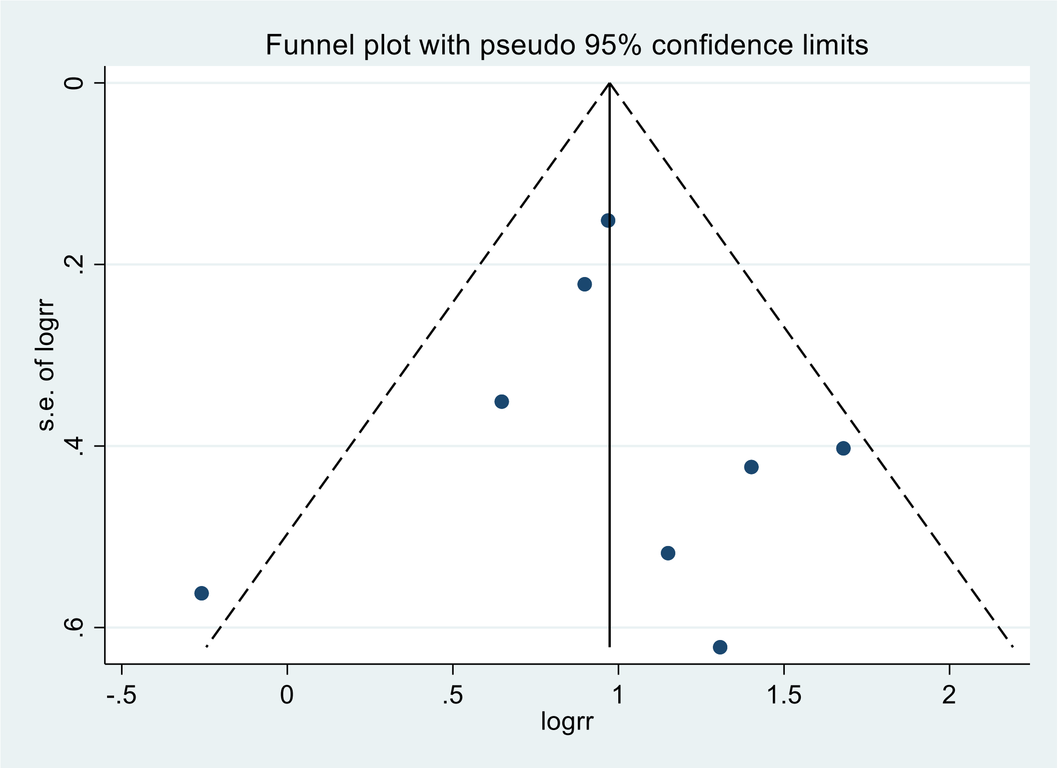

Figure S8. Funnel plot with pseudo 95% confidence intervals with included studies on Figure S1.

Results

On full-text assessment, we excluded six studies due to inappropriate study design or inappropriate inclusion criteria. Exclusion criteria from the qualitative synthesis included: one previous meta-analysis, one review that assessed the rate of FLD in AP patients, two studies reported only on severe FLD (defined by hepatic attenuation index - HAI<0.5) cases and one case-report. A study that utilized the Nationwide Inpatient Sample database of the United States of America to assess the association between NAFLD and AP severity was also excluded because the un-proportionally low rate of NAFLD cases.

We could not include two articles in quantitative synthesis because of a lack of data. Only one study reported on long-term outcomes and one on hospital readmission.

Details of the parameters included in multivariate analysis in the included articles are summarized in Table S3.

Risk of bias assessment between studies

Based on our analysis Hao YM [3], Wang S. et al. [11] and Satapathy S. et al. [9] showed the worse results with having multiple moderate and high-risk domains. The domain of "study participation" was the best-rated, as only one study carried a high and two studies carried moderate risk of bias. In contrast, the domain of 'study confounding' was the worst rated, since multiple studies did not report how the important confounders were accounted for and whether an appropriate method was used for handling missing data.

References

1. Liberati, A.; Altman, D.G.; Tetzlaff, J.; Mulrow, C.; Gøtzsche, P.C.; Ioannidis, J.P.A.; Clarke, M.; Devereaux, P.J.; Kleijnen, J.; Moher, D. The PRISMA statement for reporting systematic reviews and meta-analyses of studies that evaluate healthcare interventions: explanation and elaboration. *BMJ* **2009**, *339*, b2700, doi:10.1136/bmj.b2700.
2. Dou J., C.N. The effect of nonalcoholic fatty liver disease on the severity of acute pancreatitis. *J Prac Med* **2017**, *33*, 3563-3565, doi:10.3969/j.issn.1006-5725.2017.21.016.
3. Hao, Y.M.; Feng, Q.X.; Feng, X.Y.; Yu, P.F.; Bai, B.; Qiu, Z.Y.; Wang, Q.; Wang, S.Q.; Zhao, Q.C. Combine identifying hepatic steatosis with APACHE-II score improves the prediction of severe acute pancreatitis. *J Dig Dis* **2015**, *16*, 60, doi:10.1111/17512980.12277.
4. Jasdanwala, S. NAFLD Diagnosed with Abdominal Ultrasound is a Marker of Severity in Acute Pancreatitis. *J Gastrointest Dig Syst* **2015**, *5*, 2, doi:10.4172/2161-069x.1000293.
5. Jia, J.; Wu, Q.; Kou, J.; Yang, M. Relationship between Fatty Liver and Pancreatitis. *Int J Clin Med* **2018**, *09*, 243-248, doi:10.4236/ijcm.2018.94021.
6. Mikolasevic, I.; Orlic, L.; Poropat, G.; Jakopcic, I.; Stimac, D.; Klanac, A.; Carovic, F.; Milic, S. Nonalcoholic fatty liver and the severity of acute pancreatitis. *Eur J Intern Med* **2017**, *38*, 73-78, doi:10.1016/j.ejim.2016.10.019.
7. Morel-Cerda Eliana C., V.-R.V.J.A., Álvarez-López Francisco, García-Jiménez Edgar S., Rangel-Orozco María F., González-Álvarez Roberto, Flores-Mendoza José F., Zaragoza-Scherman Carmen F., Velarde-Chávez José A., Mora-Huerta José A., Lazcano-Becerra Monserrat, Briones-Govea David, Pelayo Vélez Cristian O., Ledesma-Vizcarra Fernanda M., Aldana-Ledesma Juan M. Prevalence of fatty liver in patients with acute pancreatitis. *Revista Medica MD* **2019**, 10(12):113-118pp.
8. Ze-hua, P.; Lin, B.A.I.; Hong, P.U.; Long-lin, Y.I.N.; Jia-yuan, C.; Jin, J.; Ning, A.N. Abdominal CT scan in predicting complications of acute pancreatitis. *Chinese journal of general surgery* **2012**, *27*, 789-793.
9. Satapathy, S.; Friedman, B.; Bittman, M.; Aronson, S.; Kwak, N.; Novak, S.; Inamdar, S.; Cerulli, M.; David, B. Hepatic steatosis a novel marker for severe outcomes in patients with acute pancreatitis. *Am J Gastroenterol* **2011**, *106*, S115-S116, doi:10.1038/ajg.2011.336_5.
10. Suchsland, T.; Aghdassi, A.; Kühn, K.; Simon, P.; Lerch, M.M.; Mayerle, J.; Flessa, S. Predictive factors for and incidence of hospital readmissions of patients with acute and chronic pancreatitis. *Pancreatology* **2015**, *15*, 265-270, doi:10.1016/j.pan.2015.03.008.

11. Wang, S.; Zhang, X.; Li, S.; Feng, Q.; Feng, X.; Zhao, Q. Fatty liver indicates increased severity of acute pancreatitis. *J Gastroenterol Hepatol* **2013**, *28*, 240, doi:10.1111/jgh.12363_2.
12. Wu, D.; Zhang, M.; Xu, S.; Wu, K.; Wang, N.; Wang, Y.; Wu, J.; Lu, G.; Gong, W.; Ding, Y., et al. Nonalcoholic Fatty Liver Disease Aggravated the Severity of Acute Pancreatitis in Patients. *Biomed Res Int* **2019**, *2019*, 9583790, doi:10.1155/2019/9583790.
13. Xiao, B.; Zhang, X.M.; Jiang, Z.Q.; Tang, W.; Huang, X.H.; Yang, L.; Feng, Z.S. Fatty liver in acute pancreatitis: characteristics in magnetic resonance imaging. *J Comput Assist Tomogr* **2012**, *36*, 400-405, doi:10.1097/RCT.0b013e31825977c2.
14. Xu, C.; Qiao, Z.; Lu, Y.; Zhang, D.; Jia, Z.; Zhuang, X.; Shi, Y.; Xu, T.; Xing, L.; Shen, J. Influence of Fatty Liver on the Severity and Clinical Outcome in Acute Pancreatitis. *PLoS One* **2015**, *10*, e0142278, doi:10.1371/journal.pone.0142278.
15. Yoon, S.B.; Lee, I.S.; Choi, M.H.; Lee, K.; Ham, H.; Oh, H.J.; Park, S.H.; Lim, C.H.; Choi, M.G. Impact of Fatty Liver on Acute Pancreatitis Severity. *Gastroenterol Res Pract* **2017**, *2017*, 4532320, doi:10.1155/2017/4532320.
16. Yuan, L.; Tang, M.; Huang, L.; Gao, Y.; Li, X. Risk Factors of Hyperglycemia in Patients After a First Episode of Acute Pancreatitis: A Retrospective Cohort. *Pancreas* **2017**, *46*, 209-218, doi:10.1097/mpa.0000000000000738.
17. Morel-Cerda, E.C.; Velarde-Ruiz Velasco, J.A.; Álvarez-López, F.; García-Jiménez, E.S.; Rangel-Orozco, M.F.; González-Álvarez, R.; Flores-Mendoza, J.F.; Zaragoza-Scherman, C.F.; Velarde-Chávez, J.A.; Mora-Huerta, J.A. Prevalence of fatty liver in patients with acute pancreatitis. *Revista Médica* **2019**, *9*, 113-118.
